# Autologous full-thickness skin in the repair of complex ventral hernias: an innovative step into the future of complex hernia repair?

**DOI:** 10.3389/fsurg.2023.1301702

**Published:** 2023-12-15

**Authors:** Viktor Holmdahl, Karin Strigård, Ulf Gunnarsson

**Affiliations:** Department of Surgical and Perioperative Sciences, Surgery, Umeå University, Umeå, Sweden

**Keywords:** ventral hernia, parastomal hernia, incisional hernia, full-thickness skin graft, synthetic mesh

## Abstract

The repair of complex ventral hernias, such as giant incisional or parastomal hernia, is associated with a high risk for complications and recurrence. Some serious complications are related to implantation of synthetic mesh as reinforcement material. Autologous full-thickness skin graft (FTSG) as reinforcement material in the repair of these complex hernias may offer a safe alternative. This is a review of the history of FTSG use in hernia surgery and the experiences of our research group regarding its application over the last decade. The results of FTSG used in the repair of giant ventral hernias are promising, and this method may already be recommended in selected cases. We have also conducted a translational chain of preclinical studies, based on a murine model, to gain a greater understanding of the behaviour of FTSG implanted in various positions in the abdominal wall. The use of intraperitoneal FTSG as reinforcement material in parastomal hernia repair is currently being evaluated in a randomised, controlled, multicentre study.

## Introduction

Ventral hernias are common and often cause significant suffering for those patients affected. Many patients need surgical repair, and more than 8,000 ventral hernia repairs are performed each year in Sweden with a population of 10 million. Recent research into abdominal wall surgery has led to better treatment results, especially after inguinal and small ventral hernia repairs. However, repair of large ventral hernias and the subgroup parastomal hernias remains a challenge, and is associated with high risk for complications and recurrence (1, 2). It is currently recommended that repair of large ventral hernias and parastomal hernias includes some form of synthetic mesh reinforcement to reduce recurrence rates. Unfortunately, implanted synthetic mesh are sometimes associated with serious complications such as fistula formation, mesh infection, and erosion of the bowel (3). By avoiding the use of foreign materials such as a synthetic mesh, especially in complex cases of large ventral hernias and parastomal hernias, the complication rate could potentially be reduced. Full-thickness skin grafts (FTSG) as reinforcement material in the repair of ventral hernia may offer a safe alternative.

This review covers the literature on autologous skin grafting in hernia surgery and presents our more than a decade's experience of its application, from both the clinical and translational approach. The databases MEDLINE and PubMed Central was searched for relevant literature using the terms: “hernia repair”, “ventral hernia”, “parastomal hernia”, “autologous”, “cutis graft”, “skin transplantation”, “full-thickness skin graft”.

## History

The first report of autologous skin being used as reinforcement material in hernia surgery was published by Loewe in 1913, shortly followed by Rehn in 1914 (4, 5). They both described experiments where they transplanted autologous skin instead of fascia and tendon transplants, with generally good results. These pioneer studies included numerous applications of transplanted skin, including sutures, tendon repairs, and hernioplasties. In the years that followed, several studies were published on the use of skin grafting in hernia repair with results comparable to those of today. Uihlein reviewed the results of 49 incisional hernia patients operated by Rehn using autologous skin as reinforcement material, showing a recurrence rate of 12% after 2–9 years follow-up (6). In a non-randomised study on inguinal hernia repairs by Mair, FTSG was compared with the classic Bassini procedure, herniotomy, and fascial repair. The FTSG-group had superior results with recurrence rates of 0 and 1.1% (direct and indirect respectively) (7). Eisele et al. and Shaffer also presented a case series of various hernia repair types (even diaphragmatic hernias using FTSG placed intraperitoneally) where transplanted skin was employed with good results (8, 9). Experiments have also been performed on the use of FTSG in the treatment of full-thickness abdominal wall defects such as omphalocoele (10).

### Cutis grafts vs. FTSG

Early authors used cutis grafts i.e., where everything but the dermis was removed from full-thickness skin. The epidermis was removed sharply with a knife, razor, or a specially designed dermatome. The reason for this was the fear of dermoid cyst formation which was believed to arise from epidermoid tissue. Assessment of the metamorphosis of implanted autologous skin was addressed by Peer and Paddock 1937 and Peer 1939 (11, 12). They studied the histology of implanted cutis grafts and FTSG, respectively, in patients undergoing rhinoplasty for saddle nose with rib grafting. The surplus rib graft cartilage was stored subcutaneously in the chest wall of the patient and removed if no infection of the rhinoplasty occurred. This enabled the authors to implant cutis grafts and FTSG adjacent to the stored rib graft and these were removed for analysis after predefined periods of time at intervals up to 28 months postoperatively. They found that even with meticulous care traces of epidermis initially always remained in the graft, and even when completely left in place, the epidermis is still degraded and undetectable by 10 weeks. Greene et al. and Shaffer stated that the most important measure to reduce the risk of cyst formation is to suture the graft under tension (9, 13).

### Autologous skin grafting today

After the introduction of modern synthetic mesh materials, interest in research on autologous skin grafting faded, especially after the paradigm shift following studies on the Marlex™-mesh by Usher in 1959 (14, 15)*.* Synthetic mesh reinforcement predominates in recommended techniques for abdominal wall hernia repair and the results of less complex hernia repairs are satisfactory (16, 17). However, the need for alternative reinforcement materials has become evident with the increasing awareness of chronic postoperative pain and discomfort, and troublesome complications related to implanted foreign material in certain ventral hernia subgroups. Furthermore, mesh-related complications have led to patient associations and other organisations opposing the use of synthetic mesh, and several lawsuits against mesh developers have been raised (18, 19). Biological graft materials were partly developed to address the drawbacks of synthetic mesh, especially in infected cases. These biological grafts or meshes are collagen-rich plates originating from animal tissues or human cadavers. However, results using biological prostheses have been disappointing with high recurrence rates despite their considerable cost (20). As a result, interest in autologous skin grafting has received new attention with an increasing number of studies being published (21–25).

## Incisional hernia

Incisional hernia is common after abdominal surgery with rates around 10% after major abdominal procedures (26). The complexity of repair and the risk for complications are related to the aperture size of the fascial defect, BMI, comorbidity, and others (27). In view of this, and after a proof-of-concept publication, our research group conducted a feasibility study using FTSG in the onlay position in eight high-risk patients with a giant (defined as a hernial aperture >10 cm, EHS classification W3) ventral hernia (28, 29). Results were promising, with only one recurrence after an average of 33 months. To properly evaluate the use of FTSG in incisional hernia repair, a randomised controlled trial was initiated comparing onlay plasty using FTSG with synthetic mesh repair, using the best possible placement. A total of 52 patients with a giant incisional hernia were included and the primary outcome of the trial was short-term surgical complication rate. All FTSG could be harvested from the midline incision, so no additional incisions had to be made. However, FTSG harvesting has been performed from the thigh and the upper arm on patients not included in this RCT without any significant donor site morbidity. At a 3-month follow-up, no difference in numbers of complications was noted despite differences in positioning of the graft. Patients in the FTSG group, however, described less abdominal wall pain (30). At a 1-year follow-up, besides a clinical assessment, participants underwent Biodex™ testing to detect any change in abdominal wall muscle strength. No differences in abdominal wall muscle strength or recurrence rate were seen between the groups (31). At 3 years, a long-term follow-up was carried out including a clinical visit and an assessment of the study participants' quality-of-life using validated questionnaires VHPQ, EQ-5D, and SF-36. Quality-of-life assessment was also carried out by mail after an average of 9 years and compared to values attained prior to surgery. In general, patients reported improved quality-of life, but there were no significant differences in quality-of-life or any other clinical outcome measure (recurrence rate, long-term complications etc.) between the groups. The trial thus indicated that FTSG is a valid alternative to conventional synthetic mesh in the repair of giant ventral hernias.

## Parastomal hernia

Having an enterostomy often decreases the patient's quality-of-life, and stomal complications increase this burden (32). Parastomal hernia is a common complication of an enterostomy, affecting up to 80% depending on the definition and diagnostic modality employed (33–37).

A complicating factor in parastomal hernia repair is that the fascial aperture can only be repaired partially to leave room for the stomal intestine. This risks aperture widening, and eventually prosthetic material must be placed adjacent to the bowel. The European Hernia Society guidelines on the treatment of parastomal hernia recommends a synthetic mesh plasty (38). However, these guidelines lack details regarding mesh positioning, mesh material, and surgical approach. Their conclusion is that there a paucity of knowledge and a need for high-quality evidence. Recurrence rates after synthetic mesh repairs are still high, as high as 46% in some studies (2, 3, 39). Furthermore, placement of a foreign body adjacent to, or against the bowel can give rise to serious and sometimes fatal complications such as bowel erosion, mesh infection, and fistula formation. These serious complications are reflected in a 6.3% 30-day mortality rate after parastomal hernia repair reported in a nationwide register study from Denmark (1). There are indications that poor parastomal hernia repair results has led to a reluctancy to treat these patients, and only 71 procedures were performed in Sweden between 1998 and 2007 (40). The lack of a gold standard for parastomal hernia repair remains a medical issue and there is an urgent need for better and safer methods of repair. Autologous FTSG offers an alternative reinforcement material that could potentially reduce the risks associated with synthetic mesh.

### Preclinical investigations

Previous studies on FTSG in hernia surgery have generally had the transplant placed in an onlay position. From our current experience of parastomal hernia repair, it seems that the optimal position for the reinforcement material is intraperitoneal, and very little has been published on intraperitoneal FTSG. Intraperitoneal placement would also lend itself to laparoscopic adaptation in the future. To increase our knowledge on the biological behaviour of FTSG prior to its use as reinforcement in parastomal hernia repair, our group designed and performed four translational research studies (41–44).

In a murine model, 20 mice received FTSG from isogenetic luciferase gene-positive donors, 10 in the onlay position and 10 in the intraperitoneal position. At fixed intervals, graft survival was evaluated *in vivo* using luminescence until the mice were sacrificed at 8 weeks. Graft survival was 100% in both groups and no FTSG-related complication was observed (41). A secondary outcome was intra-abdominal adhesions, only three of the mice in the intraperitoneal group showed low-grade adhesions (Jenkins' scale 1–2). General histology of the graft was similar in both onlay and intraperitoneal mice, and 50% of the grafts were well incorporated into the host site regardless of position, with signs of tissue remodelling, inflammation, degradation of adnexa, and microvascular networks (42). In a subsequent study on the same mice population, extracellular matrix components such as collagen types I, III, IV, V and their endogenous inhibitors matrix metalloproteinases 1, 8 and 9 were studied by histology and immunohistochemistry (43). No noticeable differences in these parameters were evident between grafts in the intraperitoneal and the onlay positions. The promising results from this murine model paved the way for testing its intraperitoneal use in humans as well as in the previously tested onlay position.

To further explore the properties of human FTSG, an experimental model was designed to evaluate the mechanical properties of FTSG (44). Fresh FTSG was gathered during surgical procedures from patients with a wide range of backgrounds. The skin samples were then tested for tensile strength and suture load. Tensile strength was tested in a specially designed test frame using a spring-loaded dynamometer. Identical tests were then performed on a synthetic mesh (Symbotex™) and a biological mesh (XenMatrix™). The FTSG demonstrated a mean tensile strength of 604 N/cm which was significantly higher than both the synthetic mesh (40 N/cm *p* = 0.0007) and the biological mesh (208 N/cm *p* = 0.0005). Suture load limit was tested by passing a suture through the specimen and applying traction until either the tissue/mesh or the suture gave way. The FTSG resistance to suture load was as high or higher than the meshes (67 N vs. 63 N (*p* = 0.0687) and 30 N (*p* = 0.0007) respectively).

From the results of these translational studies, we concluded that we have enough evidence to proceed with developing a method to repair parastomal hernia using FTSG as reinforcement in the intraperitoneal position. Our method was described and tested in a feasibility study on four patients published in 2021 (45). In short, the method involves a midline laparotomy where the FTSG is harvested from the midline incision. The stoma is dissected from the skin and retracted into the abdominal cavity. The hernia aperture is reduced to an adequate size depending on the type of stoma and the FTSG is applied around the stomal intestine and sutured to the intestine and abdominal wall along edges of the graft. After a mean follow-up of 18 months, no major procedure-related complication was noted.

### SHIFT

The promising results of the feasibility study encouraged us to evaluate the new method in a larger setting. In December 2019, we began enrolling patients to the SHIFT study (Stoma Hernia Intraperitoneal Full-Thickness skin); a randomised, controlled, multicentre study where FTSG is compared to Dynamesh IPST™ as reinforcement material in parastomal hernia repair. The study protocol has been published in BMC Trials and is on clinicaltrials.gov (ID no: NCT03667287), and enrolment is ongoing (46). The main outcome of the study is surgical complication 3 months postoperatively. Secondary outcomes of the trial are recurrence, quality-of-life [measured with ventral hernia pain questionnaire (VHPQ) and EQ-5D], and healthcare economy. The study will include clinical follow-ups at 3, 12, and 36 months postoperatively.

### Biology of the abdominal wall

Ventral hernias develop through a complex interaction of endogenous and exogenous factors and differ significantly regarding pathogenesis and prognosis. Despite this, clinical presentations are similar. Interest in the role played by tissue biology and connective tissue metabolism has increased in recent years. Imbalances between matrix metalloproteinases and their endogenous inhibitors could predispose to hernia development (47). However, little is known about the interplay between connective tissue composition and metabolism and its relation to hernia disease. As a translational component of the SHIFT study, we will collect tissue samples from muscle, fascia, and skin during the primary procedure. We will also draw blood samples preoperatively, Day 1 postoperatively, and at the clinical follow-ups. The tissue samples will allow us to investigate connective tissue collagen composition, hyaluronic acid, and inflammatory activity. Consecutive blood samples will also give us a dynamic insight into connective tissue turnover and wound healing during the postoperative healing phase.

There is little known about the fate of grafts used in hernia surgery after implantation, and most systematic investigations are from industry-financed animal studies. Since commercially available biological meshes and autologous FTSG will undergo some form of metamorphosis after implantation, we aim to obtain samples of the reinforcement material at least 1 year after surgery. We will therefore invite a subgroup of patients (*n* = 12) randomised to the FTSG-group to undergo ultrasound-guided biopsy of the implanted graft *in situ* under local anaesthesia. This will allow us to investigate the histology and microenvironment of the implanted tissue and how it has been transformed over approximately 1 year.

## Discussion

FTSG is autologous living tissue that can easily adapt to its donor site. The histological investigations by Peer and Paddock revealed complete degradation of the adnexa of implanted skin, leaving only dense irregular connective tissue, similar to fascia, which correlates well with our own results (11, 12, 42, 48). Implantation of an FTSG in the abdominal wall will therefore result in the deposition of a large amount of collagen to reinforce weakened structures without having to implant foreign material.

FTSG undergoes remodelling and metamorphosis after implantation and little is known about this process. However, this transformation could be its advantage over other reinforcement materials (11). Since it is autologous, FTSG has the potential to fully integrate with abdominal wall tissues rather than depending on the scarring process induced by the foreign body reaction to synthetic mesh materials. Patients with a giant ventral hernia who received a FTSG reported less postoperative discomfort than patients receiving a synthetic mesh. This may indicate better tissue integration and better abdominal wall compliance (48). The aim of hernia surgery is to reduce symptoms and restore abdominal wall function, and there are possible advantages of using reinforcement material that integrates with the abdominal wall such as FTSG, rather than an inert inflexible synthetic mesh. This is especially important in cases with a large hernial defect where a mesh covering a significant proportion of the abdominal wall is often required. Furthermore, integration of the FTSG in parastomal hernia repair could enable the graft to become a continuum between the abdominal fascia and the stomal intestine. This continuum could prevent recurrence adjacent to the stomal intestine and may also prevent prolapse of the intestine.

There are several reasons why FTSG could be superior to synthetic meshes and biological prosthetics. One reason lies in the process of vascular ingrowth. Since biological prosthetics are xeno- or allografts, they are decellularised in various ways to prevent transplant rejection. This bioavailability treatment enables the specimen to act as an extracellular matrix scaffold, so that host cells can colonise and regenerate. The result is functional replacement of the defect tissue that caused the hernia, but the process is dependent on the host's rather slow neovascularisation of the graft. Autologous FTSG, however, is a living tissue with pre-existing blood vessels. By a process called inosculation, the vasculature at the host site integrates with the FTSG's already existing vasculature (49). This leads to reperfusion of the graft's vasculature within a few days, thus preventing graft necrosis. The transplantation of autologous skin also provides a graft with a pre-existing immunological infrastructure, possibly making them more resilient to infection.

## Conclusion

The need for an alternative strategy in the repair of complex ventral hernias is evident. Autologous FTSG could be the phoenix of complex hernia surgery. Our research group has now gained more than a decade of experience of its use in complex ventral hernia surgery, and this has encouraged us to proceed with further investigations. The results of our trial on giant ventral hernia patients where FTSG was comparable to conventional mesh repair implicates the possibility for its use in selected cases. One future area of research is to adapt the method for laparoscopic repair. Much more research is necessary before FTSG can be widely introduced in hernia surgery. We hope that this review will stimulate further research on the use of FTSG in hernia surgery, to potentially offer an additional tool in the hernia surgeons toolbox.
